# One new species and three new records of *Chrysis* Linnaeus from China (Hymenoptera, Chrysididae)

**DOI:** 10.3897/zookeys.669.12398

**Published:** 2017-04-21

**Authors:** Paolo Rosa, Na-sen Wei, Zai-fu Xu

**Affiliations:** 1 Via Belvedere 8/d, I-20881 Bernareggio (MB), Italy; 2 Department of Entomology, College of Agriculture, South China Agricultural University, Guangzhou 510640, China

**Keywords:** *Chrysis*, *
antennata* species-group, *
capitalis* species-group, *
elegans* species-group, *
maculicornis* species-group, new species, new records, China

## Abstract

Four Chinese *Chrysis* species-groups, the *antennata*, *capitalis*, *elegans*, and *maculicornis* species-groups, are discussed. *Chrysis lapislazulina* Rosa & Xu, **sp. n.** is described in the *elegans* species-group; and three species, *C. brachyceras* Bischoff, 1910, *C. subdistincta* Linsenmaier, 1968 and *C. yoshikawai* Tsuneki, 1961, are reported for the first time from China in other species-groups. A new synonymy is proposed for *C. ignifascia* Mocsáry, 1893 = *C. taiwana* Tsuneki, 1970, **syn. n.** A short historical review of the *elegans* species-group is provided. *C. goetheana* Semenov, 1967 is transferred from the *elegans* species-group to the *maculicornis* species-group. *C. mesochlora* Mocsáry, 1893 is considered a *nomen dubium*.

## Introduction

Kimsey and Bohart (1991) provided keys and detailed diagnoses for the identification of *Chrysis* species-groups from all zoogeographical regions. Their classification and characterization of species-groups is adopted here with few exceptions (Rosa et al. 2014). However, some species-groups are currently under investigation; in particular, the *antennata* species-group which is more closely related to the genus *Praestochrysis* Linsenmaier, 1959 than to the genus *Chrysis*.

At present, there are 79 known species of Chinese *Chrysis* (Rosa et al. 2014, 2016a); but this genus needs to be more intensively investigated (Rosa et al. 2016a). Many Chinese chrysidid specimens have been collected over the last twenty years and some of the main findings have been published (Rosa et al. 2015a, 2015b, 2016a). In the present paper four *Chrysis* species-groups are discussed, namely the *antennata*, *capitalis*, *elegans*, and *maculicornis* species-groups. A new species is also described, *C. lapislazulina* sp. n. belonging to the *elegans* species-group, and three new records from China are reported: *C. brachyceras* Bischoff, 1910 in the *antennata* species-group, *C. yoshikawai* Tsuneki, 1961 in the *capitalis* species-group, and *C. subdistincta* Linsenmaier, 1968 in the *maculicornis* species-group.

## Materials and methods

All specimens were examined using a Leica MZ125 stereomicroscope. Photographs of specimens from South China Agricultural University (SCAU) were taken by a digital camera (CoolSNAP) mounted to a Zeiss Stemi 2000-CS stereomicroscope. All images were processed using Image-Pro Plus software. Photographs of the holotype of *C. taiwana* were taken with a Keyence microscope. Photographs of types from other museums were taken by a Nikon D-80 mounted on a Togal SCZ stereomicroscope and stacked through the software Combine ZP.

Terminology mostly follows Kimsey and Bohart (1991). Abbreviations used in the descriptions are as follows:

**BOL** the shortest distance between mid-ocellus and transverse frontal carina (TFC);

**F1 **, **F2**, **F3**, etc. flagellomeres 1, 2, 3, etc.;

**l/w** length/width ratio;

**MOD** mid ocellus diameter;

**MS** malar space, the shortest distance between base of mandible and lower margin of compound eye;

**OOL** the shortest distance between posterior ocellus and compound eye;

**P** pedicel;

**PD** puncture diameter;

**POL** the shortest distance between posterior ocelli;

**S2** metasomal sternite 2;

**T1, T2, T3** metasomal tergites 1, 2, 3;

**TFC** transverse frontal carina.

Types and other specimens have been examined from the following institutions:

**HNHM** Hungarian Natural History Museum, Budapest, Hungary;

**MNHU** Museum of Natural History of the Humboldt-Universität, Berlin, Germany;

**MSNG** Museum of Natural History “G. Doria”, Genoa, Italy;

**NMLS** Natur Museum Luzern, Switzerland;

**OMNH** Osaka Museum of Natural History, Osaka, Japan;

**SCAU** Hymenopteran Collection, South China Agricultural University, Guangzhou, China;

**SHEM** Shanghai Entomological Museum, Chinese Academy of Sciences, Shanghai, China;

**ZISP** Zoological Institute, St. Petersburg, Russia;

**ZMUC** Zoological Museum, University of Copenhagen, Denmark.

## Taxonomy

### *Chrysis antennata* species-group

#### Chrysis antennata

Taxon classificationAnimaliaHymenopteraChrysididae

species-group

Chrysis antennata species-group: Kimsey and Bohart 1991: 328 (key), 323 (fig. 105d), 336 (fig. 109q), 337 (diagnosis), 350 (fig. 112d).

##### Diagnosis.

The *antennata* species-group is characterised by broadened antennae, short and broad face, toothed metanotum, and similar habitus to *Praestochrysis* Linsenmaier except for four teeth on T3 (Kimsey and Bohart 1991).

##### Description.

Male F1 l/w = 1.2; female F1 l/w = 1.5. Flagellomeres broadened, with F4 broader than long. TFC almost straight, slightly downcurved laterally, Mid ocellus lidded. Male MS = 1.5 MOD; female MS = 1.8–2.2 MOD. Metanotum with small, stout, postero-median tooth. T3 short, weakly saddled in female, with weak transversal prepit bulge; pit row deep; lateral margins simple; apex with four short apical teeth. Black spots on S2 round and well separated in *C. antennata*, triangular and almost fused in *C. brachyceras*.

##### Biology.

Unknown.

##### Species included.

Two species: *Chrysis antennata* Mocs*á*ry, 1912 from Afrotropical Region (Mocs*á*ry 1912a), and *C. brachyceras* Bischoff, 1910 from Oriental Region.

##### Distribution.

Afrotropical and Oriental regions.

#### Chrysis brachyceras

Taxon classificationAnimaliaHymenopteraChrysididae

Bischoff, 1910

Fig. 1

Chrysis (Tetrachrysis) brachyceras Bischoff, 1910: 474. Holotype, ♀; Malaysia (MNHU) (examined).
Chrysis (Tetrachrysis) brachyceras : Bischoff 1913: 48 (Malaysia).
Chrysis brachyceras : Kimsey and Bohart 1991: 337 (Malaysia, Laos), 390 (Malaysia).

##### Type material.

Holotype, ♀, MALAYSIA, Malacca (MNHU). **Additional material**: 5♀♀, CHINA, Guangxi, Maoershan National Nature Reserve, 3.VIII.2005, leg. Liu-sheng Chen, ANT001–ANT005 (SCAU); 2♀♀, CHINA, Guizhou, Tianzhu, VIII.2009, leg. Yang-wen Wang, ANT006, ANT007 (SCAU).

**Figure 1.**
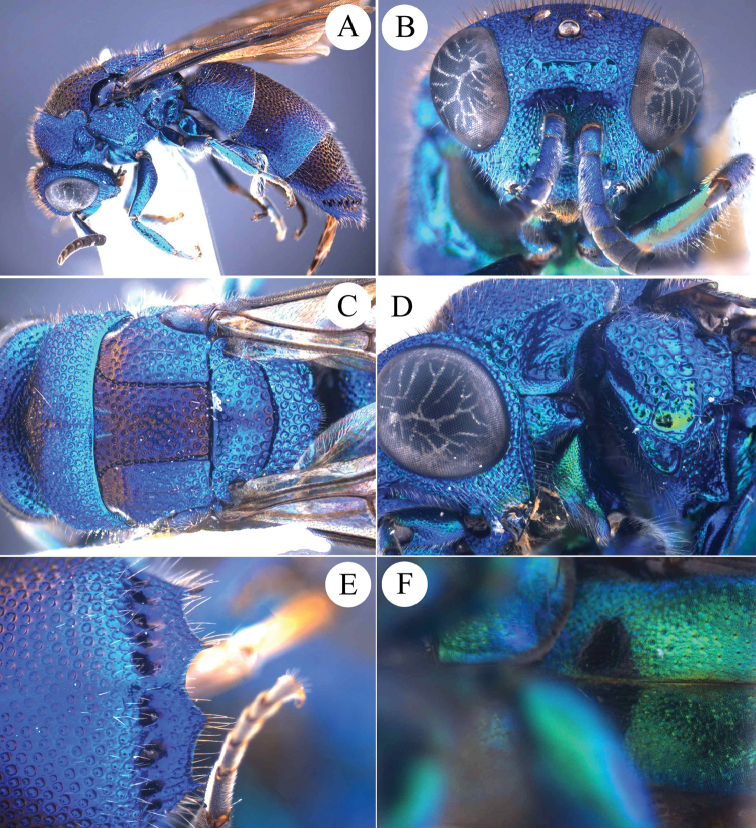
*Chrysis brachyceras* Bischoff, 1910, ♀ from Guangxi. **A** Habitus, lateral view **B** head, frontal view **C** mesosoma, dorsal view **D** head, pronotum and mesopleuron, lateral view **E** apex of T3, dorsal view **F** black spots on S2, ventral view.

##### Diagnosis.

*Chrysis brachyceras* is a large species, with the following distinctive characteristics: flagellomeres broad and flat; metanotum with small and stout postero-median tooth; T3 with four short apical teeth.

##### Description.

*Female*. Body length 10.0–11.0 mm.

*Head.* Scapal basin fully punctate. TFC medially straight, slightly downcurved at both ends and with two weak branches extending upwards (Fig. 1B). Relative length of P:F1:F2:F3 = 1.0:2.3:0.9:0.9. OOL = 1.5 MOD; BOL = 1.0 MOD; POL = 1.5 MOD; MS = 1.8 MOD; subantennal space 1.0 MOD. Anterior margin of clypeus emarginate. F3–F11 broadened and flattened. Genal carina sharp all over its length (Fig. 1D).

*Mesosoma.* Pronotal groove deep and almost reaching posterior margin; sublateral carina distinct and complete (Figs 1A, 1D); pronotal side with depression. Mesoscutum evenly punctate (Fig. 1C). Mesoscutellum simple, without anterior depression or fovea. Metanotum with a small, stout tooth pointing upwards (Figs 1A, 1C). Mesopleuron with broad episternal and scrobal sulci; the latter similar to elongate foveae (Fig. 1D).

*Metasoma.* Metasoma evenly punctate; the punctures as large as on mesoscutum. T2 and T3 without median ridge; T3 weakly saddled in female, with weak transversal prepit bulge over deep pit row; T3 with four apical teeth and simple lateral margins (Figs 1A, 1E). Black spots on S2 triangular, almost fused along the midline (Fig. 1F).

*Colouration.* Body blue, with dark blue to green metallic reflections, dark blue on ocellar area, mesoscutum medially, T1 medially, T2 and T3 antero-laterally.

*Male.* Not available for this study.

##### Distribution.

China (new record). Malaysia and Laos (Bischoff 1913; Kimsey and Bohart 1991, not Indonesia).

##### Remarks.

Kimsey and Bohart (1991) noticed some similarities between the species of the *antennata* species-group and those of the genus *Praestochrysis* Linsenmaier, 1959. The former ones are included in the genus *Chrysis* because of the four apical teeth on T3. Nevertheless, *C. brachyceras* shares with *Praestochrysis* the following characteristics: general habitus, shape of head distinctly broader than high, broadened flagellomeres, subantennal space 1.0 MOD and shorter than MS, TFC weakly indicated across strongly developed brow, scapal basin not microridged, pronotum with deep lateral depressions, metanotum with a short, stout tooth, scrobal and episternal sulci well developed and expanded ventrally, black spots on S2 small and almost fused along the midline. Several of above features (excluding broadened flagellomeres, weak TFC, metanotum with a small tooth) and pronotal sublateral carina distinct and complete are shared with the *T. lusca* species-group, which was considered belonging to the genus *Praestochrysis* by Kimsey and Bohart (1991) and *Trichrysis* by Linsenmaier (1994), Madl and Rosa (2012) and Rosa et al. (2014, 2016b). *Trichrysis lusca* is considered as belonging to *Trichrysis* not only morphologically but also biologically. *Praestochrysis* are well known parasitoids of moth prepupae (Limacodidae) (Kimsey and Bohart 1991), whereas species in the *T. lusca* species-group are parasitoids of Sphecidae (Mocsáry 1889, 1912b; Tsuneki 1955; Linsenmaier 1959) or Eumeninae (Vespidae) (Kimsey and Bohart 1991). Unfortunately, the biology of *C. brachyceras* is unknown; therefore, we consider *C. brachyceras* as a member of the genus *Chrysis* until new biological or molecular evidence is available.

### *Chrysis capitalis* species-group

#### Chrysis capitalis

Taxon classificationAnimaliaHymenopteraChrysididae

species-group

Chrysis capitalis species-group: Kimsey and Bohart 1991: 325 (key), 329 (fig. 107p), 336 (fig. 110m), 339 (diagnosis), 350 (fig. 112j).

##### Diagnosis.

The *capitalis* species-group is characterised by apex of T3 simple, TFC prominent and M-shaped, and mid ocellus lidded. Some species in the *capitalis* species-group are also easily recognised by female metasoma usually blue to green with golden stripes (e.g. *C. abuensis* Nurse, 1902, *C. bayadera* du Buysson, 1896, *C. ignifascia* Mocsáry, 1893, and *C. jalala* Nurse, 1902).

##### Description.

Scapal basin microridged medially. Male F1 l/w = 1.5; female F1 l/w = 1.9–3.0. TFC usually well developed and M-shaped. Mid ocellus lidded. MS usually < 1.0 MOD. T2 with median ridge. T3 weakly saddled in female; pit row moderately impressed; apex of T3 convex or slightly concave medially, without apical teeth.

##### Biology.

Unknown.

##### Species included.

Fourteen species: seven Afrotropical, *Chrysis capitalis* Dahlbom, 1854, *C. dalmanni* Dahlbom, 1845, *C. infuscata* Brullé, 1846, *C. jugum* Dahlbom, 1850, *C. levioris* Edney, 1952, *C. rutilata* du Buysson, 1898b, and *C. sinuosa* Dahlbom, 1845 (Rosa and Vårdal 2015); six Oriental, *C. abuensis* Nurse, *C. bayadera* du Buysson, *C. ignifascia* Mocsáry, 1893 (= *C. taiwana* Tsuneki, 1970, syn. n.), *C. sumptuosa* Smith, 1858, *C. wroughtoni* du Buysson, 1896, and *C. yoshikawai* Tsuneki, 1961; and one Palaearctic species, *C. jalala* Nurse.

##### Distribution.

Afrotropical, Oriental and Palaearctic regions.

##### Remarks.

*Chrysis arabica* Mocsáry, 1911 was moved to the newly created *C. arabica* species-group by Linsenmaier (1994).

##### Key to Chinese species of the *capitalis* species-group

**Table:** 

1	T2 with a broad transverse posterior reddish golden stripe, contrasting with the remaining body colouration (Figs 2E, 2F)	***C. ignifascia* Mocsáry (♀)**
–	T2 blue to green without reddish or golden stripe (Figs 3, 6)	**2**
2	Female and male with sub-reniform and transverse black spots on S2 (Fig. 7B)	***C. yoshikawai* Tsuneki**
–	Male with sub-triangular and longitudinal black spots on S2 (Fig. 7A)	***C. ignifascia* Mocsáry (♂)**

#### Chrysis ignifascia

Taxon classificationAnimaliaHymenopteraChrysididae

Mocsáry, 1893

**Figure 2.**
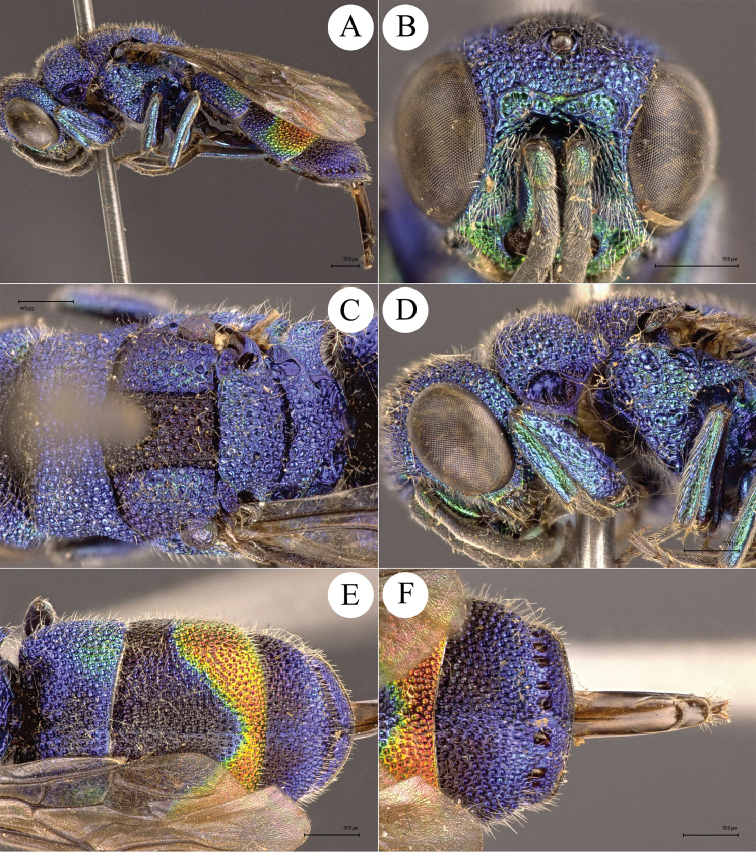
*Chrysis ignifascia* Mocsáry, 1893, ♀ from Fujian. **A** Habitus, lateral view **B** head, frontal view **C** mesosoma, dorsal view **D** head, pronotum and mesopleuron, lateral view **E** metasoma, dorsal view **F** T3, dorsal view.

**Figure 3.**
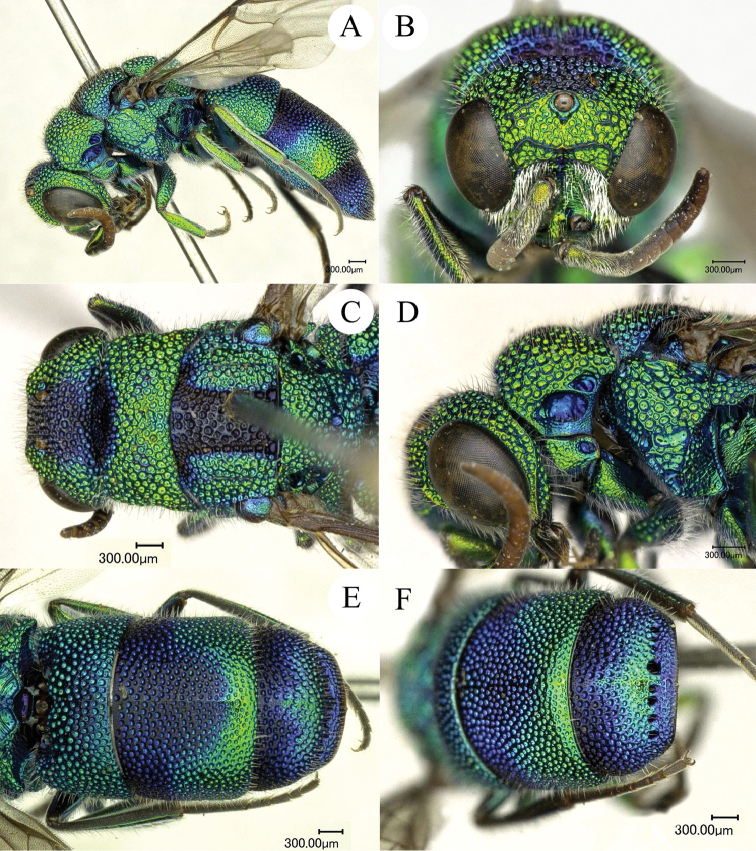
*Chrysis taiwana* Tsuneki, 1970, holotype, ♂. **A** Habitus, lateral view **B** head, frontal view **C** head and mesosoma, dorsal view **D** head, pronotum and mesopleuron, lateral view **E** metasoma, dorsal view **F** T3, dorsal view (photos by courtesy of Rikio Matsumoto, OMNH).

**Figure 4.**
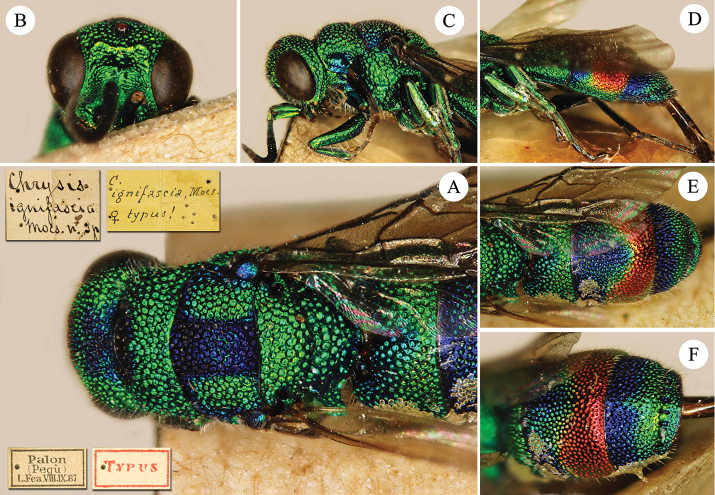
*Chrysis ignifascia* Mocsáry, 1893, holotype, ♀. **A** Head, mesosoma and T1, dorsal view **B** head, frontal view **C** head and mesosoma, lateral view **D** metasoma, lateral view **E** metasoma, dorsal view **F** T2 and T3, dorso-lateral view.

**Figure 5.**
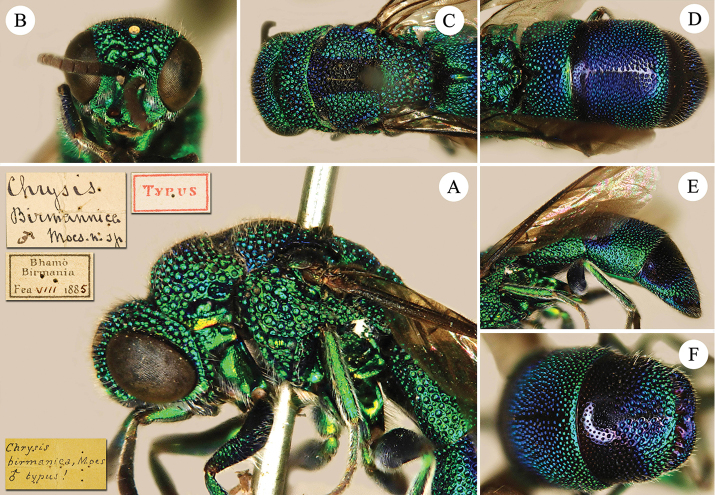
*Chrysis birmanica* Mocsáry, 1893, holotype, ♂ (= *C. ignifascia* Mocsáry). **A** Head, mesosoma and T1, lateral view **B** head, frontal view **C** head, mesosoma and T1, dorsal view **D** metasoma, dorsal view **E** metasoma, lateral view **F** T2 and T3, dorsal view.

Figs 2 , 3 , 4 , 5 , 7A

Chrysis (Holochrysis) ignifascia Mocsáry, 1893: 215. Holotype, ♀, Myanmar (MSNG) (examined). Rosa 2009: 233.
Chrysis (Holochrysis) birmanica Mocsáry, 1893: 214. Holotype, ♂, Myanmar (MSNG) (examined). Rosa 2009: 221. (Synonymised by Kimsey and Bohart 1991: 420).
Chrysis (Chrysura) taiwana Tsuneki, 1970: 7. Holotype, ♂, China (OMNH) (examined). Syn. n.

##### Type material.

Holotype, ♀, MYANMAR [Burma] Palon (Pegù), L. Fea VIII–IX.[18]87, *Chrysis ignifascia*, ♀, Mocs. n. sp. <handwritten by Mocsáry>, Typus, *C. ignifascia*, Mocs., ♀, typus! <handwritten by Mantero> (MSNG). Holotype, ♂, MYANMAR [Burma] Bhamò, Birmania, Fea VIII 1885, *Chrysis birmanica*, ♂, Mocs. n. sp. <handwritten by Mocsáry>, Typus, *C. birmanica*, Mocs., ♂, typus! <handwritten by Mantero> (MSNG). Holotype, ♂, [CHINA], Formosa [Taiwan], Pintung Hsien, Hengchun, 2.VIII.1966. leg. K. Tsuneki // Chrysis (Chrysura) taiwana Tsuneki Holotypus <handwritten> (OMNH). **Additional material**: 1♀, CHINA, Guangdong, Fogang, Guanyinshan, 15–16.IX.2007, leg. Zai-fu Xu, CAP001 (SCAU); 1♀, CHINA, Fujian, Jianning, 8.VI.1959, leg. Gen-tao Jin & Ming-yang Lin, 34022848 (SHEM); 1♀, CHINA, Taiwan, Koshun, Apr. 1937, coll. K. Iwata (NMLS); 1♂, MYANMAR, Lower Burma, Shwègyin 6.[18]98 Bingham, *Chrysis burmanica* [!], ♂, Mocs., *burmanica* [!] Mocs. det. Bingham, *Chrysis burmanica* [!] Mocs. det. Mocsáry (HNHM).

##### Diagnosis.

*Chrysis ignifascia* Mocsáry female is easily recognised by the reddish golden stripe on T2 (Figs 2E, 2F). The male is green to blue, similar to *C. yoshikawai* Tsuneki, but can be separated by the longitudinal sub-triangular black spots on S2 (Fig. 7A) (transverse and sub-reniform in *C. yoshikawai* (Fig. 7B)).

##### Distribution.

China (Fujian, Taiwan, Guangdong) (Rosa *et al* 2014), Myanmar (Mocsáry 1893; Kimsey and Bohart 1991).

##### Remarks.

The colour dimorphism between male and female of *C. ignifascia* misled some authors including Mocsáry (1893), who described the female as *C. ignifascia* (Fig. 4) and the male as *C. birmanica* (Fig. 5). Tsuneki (1961, 1970) did not mention either *C. ignifascia* or *C. birmanica* in his publications and described the male as *C. taiwana* (Fig. 3), comparing its body colouration with that of *C. yoshikawai* Tsuneki, 1961. After types examination we propose the synonymy *C. ignifascia* Mocsáry, 1893 = *C. taiwana* Tsuneki, 1970, syn. n.

#### Chrysis yoshikawai

Taxon classificationAnimaliaHymenopteraChrysididae

Tsuneki, 1961

Figs 6A–F , 7B

Chrysis yoshikawai Tsuneki, 1961: 371. Holotype, ♀, Thailand (depository?).
Chrysis yoshikawai : Kimsey and Bohart 1991: 479 (Thailand).

##### Additional material.

1♀, CHINA, Yunnan, Jingdong, Jingping, 28.IV.2005, leg. He-sheng Wang, CAP004 (SCAU); 1♀, CHINA, Yunnan, Dehong, Longchuan, 1–9.VIII.2011, leg. Ju-jian Chen, CAP005 (SCAU).

**Figure 6.**
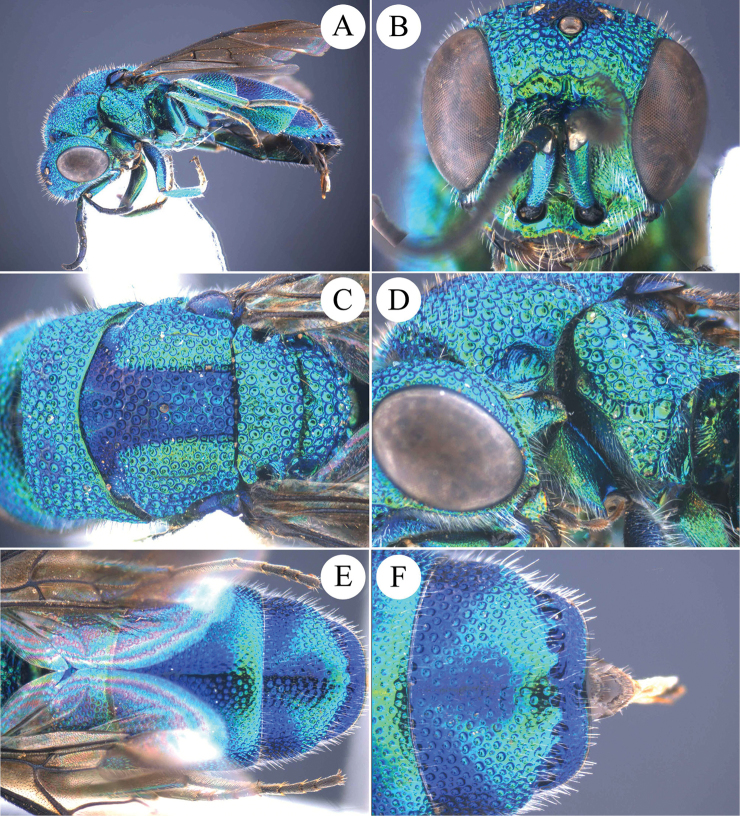
*Chrysis yoshikawai* Tsuneki, 1961, ♀ from Yunnan. **A** Habitus, lateral view **B** head, frontal view **C** mesosoma, dorsal view **D** head, pronotum and mesopleuron, lateral view **E** metasoma, dorsal view **F** T3, dorsal view.

##### Diagnosis.

**Figure 7.**
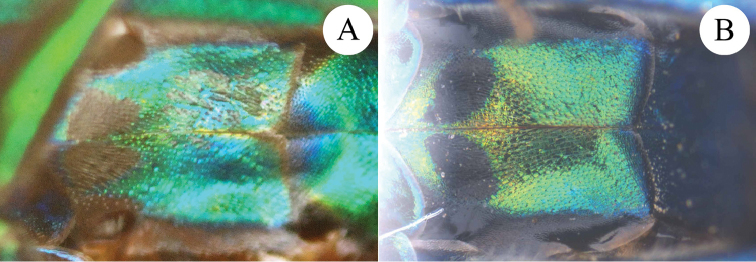
Black spots on S2 of females, ventral view. **A** *Chrysis ignifascia* Mocsáry, 1893 **B** *Chrysis yoshikawai* Tsuneki, 1961.

*Chrysis yoshikawai* is similar to *C. ignifascia*, but can be separated by: female body entirely green to blue, without reddish or golden colouration (with reddish golden stripe posteriorly on T2 in *C. ignifascia*), male S2 with sub-reniform and transverse black spots (Fig. 7B) (sub-triangular and longitudinal in *C. ignifascia*, Fig. 7A), and T3 with darkened clover-shaped spot (Figs 6E, 6F).

##### Description.

*Female* (Fig. 6A). Body length 7.5 mm.

*Head.* Scapal basin fully striate and with micropunctate ground sculpture. TFC double, sharply raised (Fig. 6B). Relative length of P:F1:F2:F3 = 1.0:1.3:1.0:0.7. OOL = 2.0 MOD; BOL = 1.5 MOD; POL = 2.1 MOD; MS = 0.5 MOD; subantennal space 0.5 MOD. Genal carina well developed throughout its length (Fig. 6D).

*Mesosoma.* Pronotal groove shallow and reaching 2/3 of pronotum length. Mesoscutum and mesoscutellum evenly punctate; metanotum with coarse punctures (Fig. 6C). Mesopleuron with deep episternal and scrobal sulci, both sulci with large foveae (Fig. 6D).

*Metasoma.* Metasoma with large, even punctures; the punctures as large as on mesoscutum (Fig. 6E). T2 and T3 with median ridge; T3 weakly saddled with deep pit row; apex of T3 slightly concave in the middle (Fig. 6F). Black spots on S2 sub-reniform, transverse, separated by less than 1.0 MOD (Fig. 7B).

*Colouration.* Body metallic blue to green, with dark blue on vertex, mesoscutum medially, tegula, T1 anteriorly, T2 anteriorly and T3 anteriorly (a typical clover-shaped pattern) and posteriorly (from pit row to posterior margin).

*Male.* Similar to female.

##### Distribution.

China (new record). Thailand (Tsuneki 1961; Kimsey and Bohart 1991).

##### Remarks.

Tsuneki (1961) did not mention the repository of the holotype. Kimsey and Bohart (1991) reported it at OMNH, wherein it was not found (Dr. Rikio Matsumoto, pers. comm.).

### *Chrysis elegans* species-group

#### Chrysis elegans

Taxon classificationAnimaliaHymenopteraChrysididae

species-group

Chrysis (Chrysis) elegans species-group: Linsenmaier 1959: 93 (key), 136 (diagnosis).
Chrysis elegans species-group: Kimsey and Bohart 1991: 325 (key), 345 (diagnosis), 329 (fig. 107d), 335 (fig. 109u), 341 (fig. 111a).

##### Diagnosis.

The *elegans* species-group is characterised by having habitus cylindrical and elongate; TFC weak or indistinct; face slightly broadened below, with subparallel and short MS; head broadened behind compound eyes in dorsal view; apex of T3 without distinct teeth, at most undulate and laterally with blunt angles; posterior margin of T3 bending downwards in females; body pubescence short and whitish; forewing radial cell closed. Body length usually 7 to 11 mm; only the North-African *C. albitarsis* is smaller (5–6 mm). Most Palaearctic species have red to golden red metasoma; females and sometimes males have mesosoma partially red. Males of *C. elegans* from eastern Mediterranean countries and Middle East can be entirely emerald green to golden green.

##### Description.

F1 l/w = 1.5–2.5. Scapal basin medially polished, especially in females. TFC weak or faint, weakly M-shaped. MS = 0.5–1.0 MOD. Pronotum longer than or as long as mesoscutellum; mesopleuron with deep scrobal sulcus. T3 pit row with small, separated pits; T3 without apical teeth, at most undulate. Black spots on S2 usually large, sometimes antero-medially fused. Male genitalia with apex of gonocoxae and cuspis considerably hirsute (Arens 2015).

##### Biology.

Members of this species-group are parasitoids of Apidae Megachilinae (Linsenmaier 1959; Kimsey and Bohart 1991).

##### Species included.

The *elegans* species-group currently includes eighteen species: *Chrysis albitarsis* Mocsáry, 1889; *C. angustifrons* Abeille de Perrin, 1878; *C. bovei* (du Buysson, 1898a); *C. castillana* (du Buysson in André, 1896); *C. deposita* Nurse, 1904; *C. dissimilis* Dahlbom, 1854; *C. eldari* (Radoszkowski, 1893); *C. elegans* Lepeletier, 1806; *C. hemera* Semenov, 1954; *C. io* Semenov, 1910; *C. joppensis* du Buysson, 1887; *C. lapislazulina* sp. n.; *C. lateralis* Dahlbom, 1845; *C. lepida* Mocsáry, 1889; *C. pushkiniana* Semenov, 1967; *C. pyrrha* Semenov, 1967; *C. rubricollis* du Buysson, 1900; *C. rueppelli* du Buysson, 1904.

##### Distribution.

Palaearctic and Oriental regions.

##### Discussion.

The *Chrysis elegans* species-group is primarily a West-Palaearctic group (Kimsey and Bohart 1991; Linsenmaier 1999; Rosa et al. 2015c), distributed from the Mediterranean basin to Middle East and central Asia, plus a new species herewith described. Only two species, *Chrysis dissimilis* Dahlbom, 1854, and *C. lapislazulina* sp. n. are known in the Oriental Region so far.

This species-group was established by Linsenmaier (1959), who originally included seven species: *C. elegans* Lepeletier, 1806; *C. angustifrons* Abeille de Perrin, 1878; *C. joppensis* du Buysson, 1887; *C. castillana* du Buysson in André, 1896; *C. ignicollis* Trautmann, 1926a; *C. separata* Trautmann, 1926a; and *C. meyeri* Linsenmaier, 1959. Later, Linsenmaier (1968) included also *C. ashabadensis* Radoszkowski, 1891 and synonymised *C. meyeri* with *C. albitarsis* Mocsáry. Kimsey and Bohart (1991) included twenty-one species, but their species-list has been partially modified in the last years: *C. albitarsis* Mocsáry which was placed into the *cuprata* species-group by Kimsey and Bohart (1991), was reintroduced into the *elegans* species-group by Linsenmaier (1999); *C. kohli* Mocsáry, 1889 was mistakenly placed into both genera *Chrysis* (*elegans* species-group) and *Pseudospinolia* Linsenmaier, 1951 (Kimsey and Bohart 1991: p. 428, sub *C. kohlii*, p. 547, as synonym of *P. marqueti* (du Buysson, 1887)), while it actually belongs to the genus *Pseudospinolia*; *C. emarginatula* Spinola, 1808 and *C. tingitana* Bischoff, 1935, both included by Kimsey and Bohart (1991) into the *elegans* species-group, are clearly separated by morphological (Linsenmaier 1959, 1999) and biological features, being parasitoids of Masarinae (Vespidae) (Linsenmaier 1968; Mauss 1996; http://www.chrysis.net/forum/) and not of Apoidea, the only known hosts of members in the *elegans* species-group (Linsenmaier 1959, 1999; Kimsey and Bohart 1991). Therefore, we follow Linsenmaier’s interpretation (1959, 1999), including these two species into the *emarginatula* species-group.

More recently, after type examination, *C. ashabadensis* was transferred into the *succincta* species-group and *C. ignicollis* was considered as a junior synonym of *C. eldari* (Radoszkowski, 1893) (Rosa et al. 2015c); *C. separata* was considered as synonym of *C. lateralis* Dahlbom (Rosa and Vårdal 2015). Arens (2015) elevated the subspecies *C. ignicollis graeca* Arens, 2004 to species rank, but in our opinion *C. graeca* is to be regarded as synonym of *C. pushkiniana* Semenov (Rosa in Arens 2015). *C. goetheana* Semenov, 1967 (whose type material has been examined at ZISP) is here transferred into the *maculicornis* species-group because of the following characteristics: male with shortened F1 and F2, female with distinct straight TFC, scapal basin entirely microridged, and MS very short.

The synonymy proposed by Trautmann (1926b), *C. cupricollis* Trautmann, 1921 = *C. rubricollis* du Buysson, 1900 is to be verified. We propose to consider *C. mesochlora* Mocsáry a nomen dubium, since the holotype of *C. mesochlora* was destroyed in Hamburg during the World War II (Kimsey and Bohart 1991), and no specimen identified by Mocsáry can be traced in his collection in Budapest or in any other European collections. Moreover, this species has never been mentioned after Mocsáry’s description, except in Kimsey and Bohart (1991).

#### Chrysis lapislazulina

Taxon classificationAnimaliaHymenopteraChrysididae

Rosa & Xu sp. n.

http://zoobank.org/82BF0F09-535A-43C4-9AD8-FEE4F5C94D8E

Figs 8 , 9

##### Material examined.

Holotype, ♀, CHINA, Yunnan, Yuxi (20°21'07"N 102°32'47"E), 20.VII.2003, leg. Qiang Li (SCAU).

##### Diagnosis.

**Figure 8.**
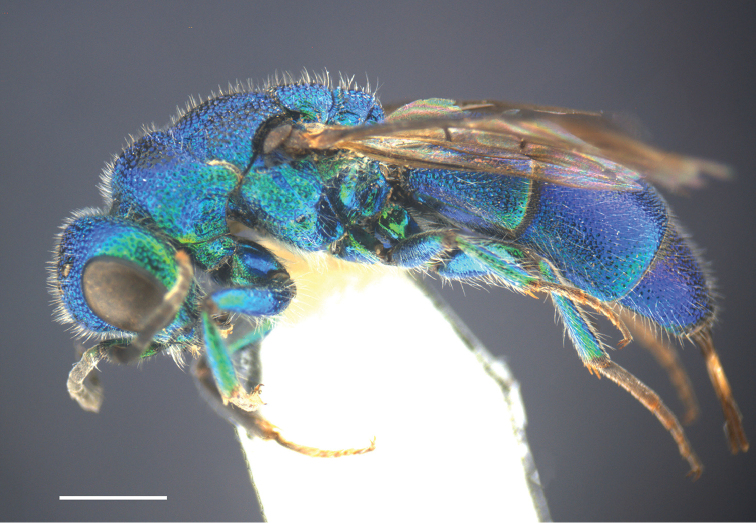
*Chrysis lapislazulina* Rosa & Xu, sp. n., holotype, ♀, habitus, lateral view. Scale bar=1 mm.

**Figure 9.**
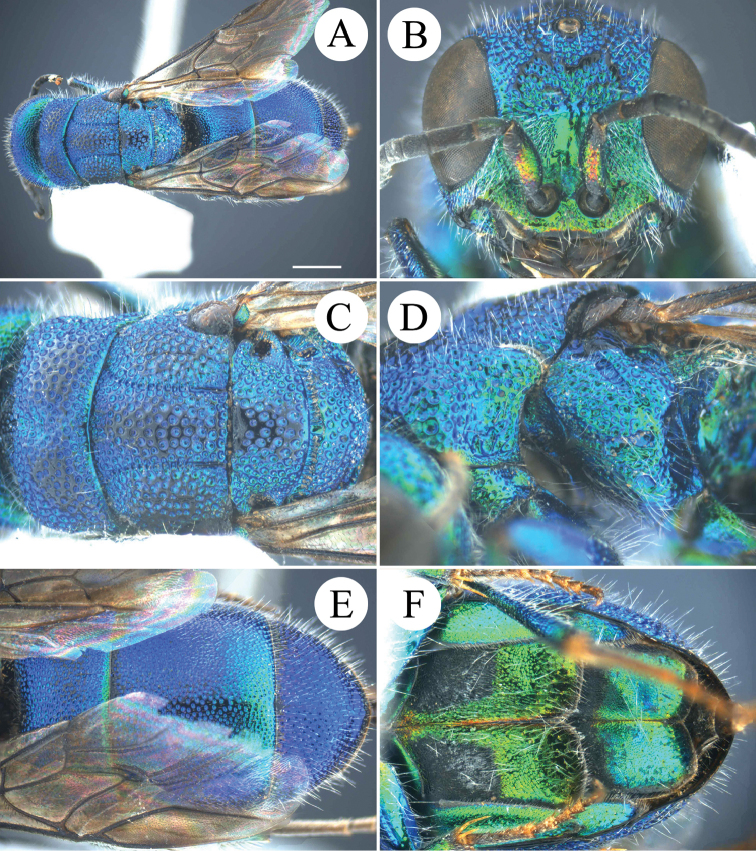
*Chrysis lapislazulina* Rosa & Xu, sp. n., holotype, ♀. **A** Habitus, dorsal view **B** head, frontal view **C** mesosoma, dorsal view **D** pronotum and mesopleuron, lateral view **E** metasoma, dorsal view **F** black spots on S2, ventral view. Scale bar 1 mm.

**Figure 10.**
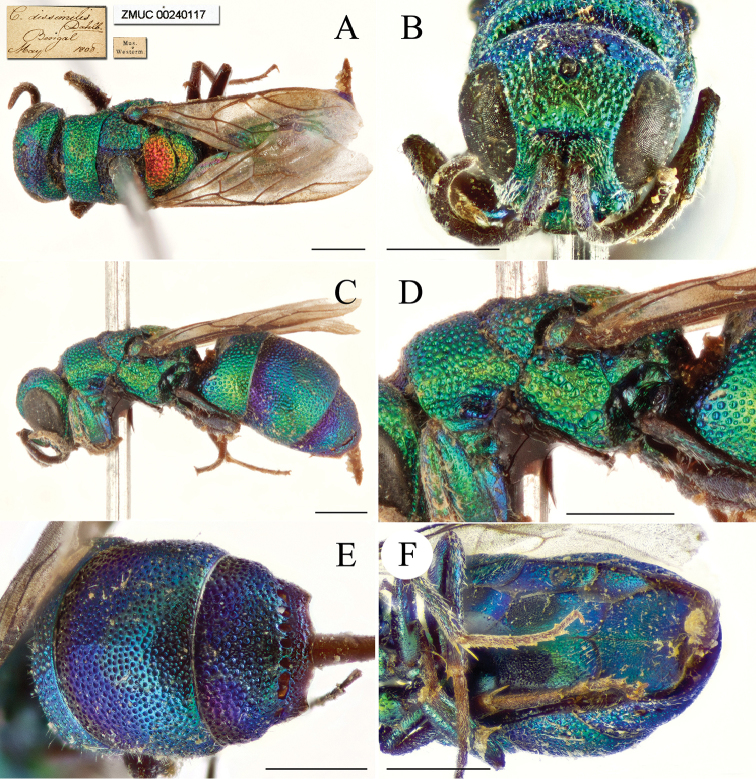
*Chrysis dissimilis* Dahlbom, 1854, holotype, ♀. **A** Habitus, dorsal view **B** head, frontal view **C** habitus, lateral view **D** pronotum and mesopleuron, lateral view **E** metasoma, dorso-posterior view **F** black spots on S2, ventral view. Scale bar 1 mm (photos by courtesy of Lars Vilhelmsen, ZMUC).

*Chrysis lapislazulina* sp. n. is recognised by the following characteristics: body blue with golden reflection (Figs 8, 9A); pronotum, mesoscutum and mesoscutellum medially with polished intervals among punctures (Fig. 9C); metasoma with fine and even punctures (Fig. 9E); apex of T3 without undulation or teeth (Fig. 9E). It can be distinguished from another Oriental species of the species-group, *C. dissimilis* by: almost uniform body colouration (mesosoma green with red scutellum and golden-red metanotum, and metasoma green with blue stripes antero-laterally on T2 and T3 in *C. dissimilis* (Fig. 10A)); apex of T3 without tooth or undulation (medially sinuous and with blunt lateral teeth in *C. dissimilis*, Figs 10C, 10E); black spots on S2 sub-rectangular (Fig. 9F) (sub-oval in *C. dissimilis*, Fig. 10F). The female of *C. lapislazulina* sp. n. can be separated from females of other Palaearctic species by: metasoma entirely blue (red to golden-red in other species); metasoma with even and fine punctures (large punctures, with or without intervals and usually decreasing in diameter posteriorly in other species); black spots on S2 sub-rectangular, basally separated by more than two MOD (vs. large and sub-oval, basally fused or narrowly separated in other species).

##### Description.

Holotype: *Female*. Body length 8.0 mm.

*Head.* Scapal basin medially polished and laterally micropunctate (Fig. 9B). TFC M-shaped, with two weak branches extending to the level of mid ocellus. Anterior margin of clypeus medially not emarginate, laterally with thickened brownish rim. Vertex with coarse punctures. Genal carina weak, present from mid gena to mandible. Relative length of P:F1:F2:F3 = 1.0:1.3:0.8:0.7; OOL = 2.0 MOD; POL = 2.3 MOD; MS = 1.0 MOD; subantennal space 1.0 MOD.

*Mesosoma.* Pronotum slightly longer than mesoscutellum (Fig. 9C); pronotal groove broad and almost reaching 2/3 of pronotum length; pronotal side with depression in dorsal view; punctuation coarse along anterior and lateral margins, with smaller punctures on pronotal groove and along posterior margin; pronotal dorsum with two darker areas with minute scattered punctures and impunctate intervals. Median lobe of mesoscutum in anterior half with broad, darker median area, with larger punctures and broader polished intervals; posterior half with even larger, contiguous, irregular punctures; lateral lobes of mesoscutum with more or less close, partly confluent punctuation; parapsidal furrow well incised. Mesoscutellum darker medially, with large punctures and broader intervals, smooth towards anterior edge; laterally with smaller, dense punctures and micropunctate intervals, punctuation reaching posterior edge. Metanotum slightly convex, with somewhat uneven punctures becoming denser postero-medially; anterior margin of metanotum with row of narrow, antero-posteriorly elongate foveae. Mesopleuron with small, shallow and round punctures, and shallow scrobal and episternal sulci (Fig. 9D).

*Metasoma.* Finely and densely punctate; punctation unusually smaller than others species of this species-group; their diameter about 1/3 to 1/4 of largest punctures on mesoscutum. T1 elongate (Fig. 9E), half as long as T2. T2 with weak or faint median ridge. T3 weakly saddled, with row of shallow small pits; apex of T3 without tooth or undulate. Black spots on S2 sub-rectangular and connected to lateral margins, widely separated medially (Fig. 9F).

*Colouration.* Body blue, darker on vertex, pronotum dorso-laterally, median and lateral lobes of mesoscutum medially (Figs 8, 9A) and mesoscutellum medially, metallic green on face and metasomal sternites (Figs 9B, 9F), with golden reflection on clypeus, scape and pedicel. Flagellomeres black (Fig. 9B). Tegula blackish brown, almost without metallic reflections. Post-tegula bright metallic blue (Fig. 9C). Forewing infuscate, with darkened anterior margin.

*Male.* Unknown.

##### Distribution.

China (Yunnan).

##### Etymology.

The specific epithet *lapislazulina* refers to the intense blue colouration with darkened areas and golden reflections; this peculiar colouration resembles the semi-precious stone lapis lazuli.

### *Chrysis maculicornis* species-group

#### Chrysis maculicornis

Taxon classificationAnimaliaHymenopteraChrysididae

species-group

Chrysis (Cornuchrysis) maculicornis species-group: Linsenmaier 1959: 173 (*partim*).
Chrysis maculicornis species-group: Kimsey and Bohart 1991: 353 (key), 341 (fig. 111m).

##### Diagnosis.

The *maculicornis* species-group is characterised by having males with shortened F1 and F2, F1 slightly longer than F2, but shorter than F3; females with F1 l/w ≈ 2.0; MS usually 0.2–1.3 MOD; face slightly wedge-shaped in frontal view.

##### Description.

Male F1 l/w = 1.0–1.4; F1 slightly longer than F2, but shorter than F3. Female F1 l/w ≈ 2.0. Scapal basin micropunctate or microridged medially. TFC well developed, biconvex. Mid ocellus sometimes lidded. MS usually 0.2–1.3 MOD. T3 in female sometimes with prepit bulge; pit row usually well developed. T3 with four sharp apical teeth. Black spots on S2 large and sub-oval, separated medially and sometimes connected to lateral margins.

##### Species included.

Kimsey and Bohart (1991) in the species-group diagnosis included fifteen Palaearctic species, but in their checklist reported twenty-two Palaearctic species, one Oriental (*C. perfecta* Cameron, 1897) and one Afrotropical (*C. rhinata* Bohart, 1988). The list of Palaearctic species needs to be further verificated, because some species have been included in the *cerastes* species-group (e.g. *C. subdistincta* Linsenmaier).

##### Distribution.

Palaearctic, Oriental and Afrotropical regions.

##### Remarks.

Linsenmaier (1959, 1968) included in the *maculicornis* species-group only the species with shortened F1 and F2 and flagellomeres yellowish beneath in males (e.g. *C. maculicornis* Klug, 1845, *C. fulvicornis* Mocsáry, 1889, and *C. stigmaticornis* Linsenmaier, 1968). Kimsey and Bohart (1991) added the species close to *C. annulata* du Buysson, 1887 (e.g. *C. blanchardi* Lucas, 1849; *C. distincta* Mocsáry, 1887; *C. rectianalis* Linsenmaier, 1968, etc.) into the *maculicornis* species-group, whereas Linsenmaier (1959, 1968) included them in the *cerastes* species-group. In this paper we follow Kimsey and Bohart’s (1991) interpretation of the *maculicornis* species-group.

#### Chrysis subdistincta

Taxon classificationAnimaliaHymenopteraChrysididae

Linsenmaier, 1968

Fig. 11

Chrysis (Chrysis) subdistincta Linsenmaier, 1968: 110. Holotype ♀; Turkmenistan (Transcaspia) (NMLS) (examined).
Chrysis subdistincta : Kimsey and Bohart 1991: 467 (*cerastes* species-group).

##### Type material.

Holotype, ♀, [TURKMENISTAN] Transcaspia Imam-baba W.Koshantschikoff // ♀ Type *Chrysis* L. *subdistincta* Lins. Linsenmaier det. 66 (NMLS). **Additional material**: 1♀, CHINA, Gansu, Jiuquan, Huangnibao, 1700 m, 15.VII.2010, leg. Xu-feng Zhang & Feng-li Cui, No. 34020575 (SHEM); 1♀, CHINA, Gansu, Jiuquan, Huangnibao, 1700 m, 16.VII.2010, leg. Xu-feng Zhang & Feng-li Cui, No. 34020062 (SHEM).

**Figure 11.**
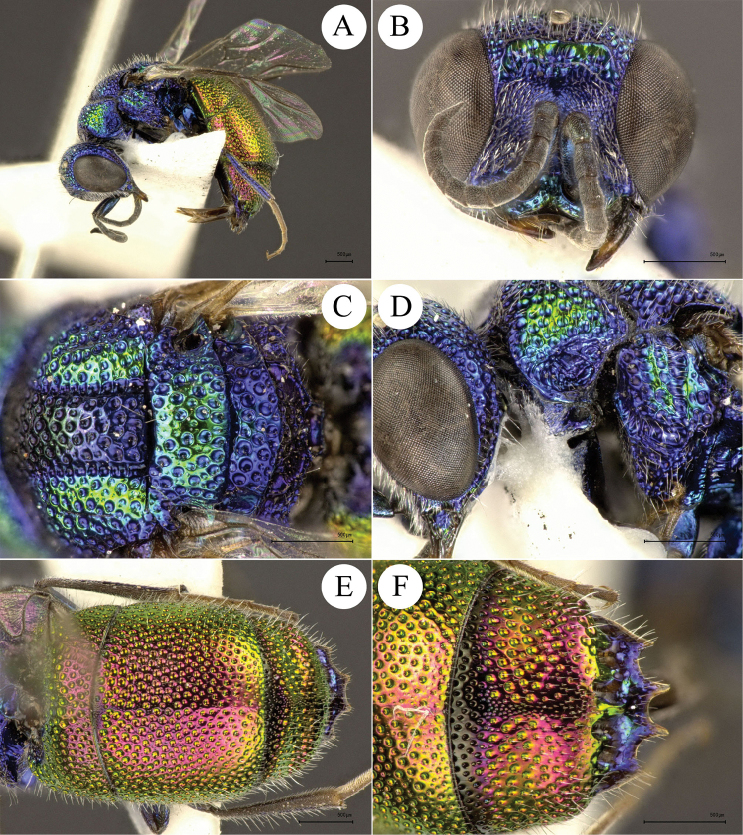
*Chrysis subdistincta* Linsenmaier, 1968, ♀ from Gansu. **A** Habitus, lateral view **B** head, frontal view **C** mesoscutum, mesoscutellum and metanotum, dorsal view **D** head, pronotum and mesopleuron, lateral view **E** metasoma, dorsal view **F** T3, dorsal view.

##### Diagnosis.

*Chrysis subdistincta* belongs to the Palaearctic *C. annulata* sub-group and is the only known Chinese species of the *maculicornis* species-group. It can be recognised by apex of T3 with median pair of apical teeth longer than lateral pair (all the apical teeth are of similar length in other species), and pit row with large, fused pits (usually small, widely separated in other species).

##### Description.

*Female* (Fig. 11A). Body length 6.5 mm.

*Head.* Scapal basin deep and micro-punctate (Fig. 11B), TFC well-developed, inverted U-shaped, with long branches in contact with eyes. Relative length of P:F1:F2:F3 = 1.0:1.2:1.0:1.0. OOL = 1.3 MOD; POL = 1.9 MOD; MS = 0.2 MOD; subantennal space 0.5 MOD. Anterior margin of clypeus broadly emarginate. Genal carina developed throughout its length.

*Mesosoma.* Pronotum medially narrowed, 0.8 times as long as mesoscutellum; pronotal groove faint. Pronotum, mesoscutum and mesoscutellum with large foveate punctures; interspaces micropunctate. Notauli with large subquadrate foveae. Mesopleuron with deep and scrobiculate episternal and scrobal sulci (Fig. 11D).

*Metasoma.* Metasoma with deep, large round punctures (Fig. 11E). PD on T2 decreasing towards posterior margin. T2 and T3 with weak median ridge. T3 pit row slightly transversely bulging before pit row; pit row with large, laterally fused pits; apex of T3 with four pointed teeth, apically hyaline, with median pair of teeth longer than lateral pair (Fig. 11F).

*Colouration.* Head and mesosoma blue, with metallic green on TFC, vertex, pronotum and mesoscutum dorso-laterally, and mesoscutellum medially. Metasoma golden to metallic reddish, with metallic blue on T3 from pit row to apical teeth.

*Male.* Unknown.

##### Distribution.

China (new record). Turkmenistan (Linsenmaier 1968).

##### Remarks.

Kimsey and Bohart (1991) followed Linsenmaier (1968) and placed *Chrysis subdistincta* into the *cerastes* species-group. Nevertheless, this species is closely related to *C. annulata* du Buysson from which it is recognizable by the elongate teeth on T3. *C. annulata* and related species have been included in the *maculicornis* species-group by Kimsey and Bohart (1991). Therefore, we consequently include *C. subdistincta* in this species-group.

## Supplementary Material

XML Treatment for Chrysis antennata

XML Treatment for Chrysis brachyceras

XML Treatment for Chrysis capitalis

XML Treatment for Chrysis ignifascia

XML Treatment for Chrysis yoshikawai

XML Treatment for Chrysis elegans

XML Treatment for Chrysis lapislazulina

XML Treatment for Chrysis maculicornis

XML Treatment for Chrysis subdistincta
